# Predicting posttraumatic stress disorder in children and parents following accidental child injury: evaluation of the Screening Tool for Early Predictors of Posttraumatic Stress Disorder (STEPP)

**DOI:** 10.1186/s12888-015-0492-z

**Published:** 2015-05-12

**Authors:** Els PM van Meijel, Maj R Gigengack, Eva Verlinden, Brent C Opmeer, Hugo A Heij, J Carel Goslings, Frank W Bloemers, Jan SK Luitse, Frits Boer, Martha A Grootenhuis, Ramón JL Lindauer

**Affiliations:** 1 Department of Child and Adolescent Psychiatry, Academic Medical Center, University of Amsterdam, Amsterdam, the Netherlands; 2 de Bascule, Academic Center for Child and Adolescent Psychiatry, Amsterdam, the Netherlands; 3 Clinical Research Unit, Academic Medical Center, University of Amsterdam, Amsterdam, the Netherlands; 4 Pediatric Surgical Center of Amsterdam, Academic Medical Center, University of Amsterdam and VU University medical center, Amsterdam, the Netherlands; 5 Trauma Unit Department of Surgery, Academic Medical Center, University of Amsterdam, Amsterdam, the Netherlands; 6 Department of Surgery, VU University medical center, Amsterdam, the Netherlands; 7 Emergency Department, Academic Medical Center, University of Amsterdam, Amsterdam, the Netherlands; 8 Pediatric Psychology Department of the Emma Children’s Hospital, Academic Medical Center, University of Amsterdam, Amsterdam, the Netherlands

**Keywords:** Posttraumatic stress disorder, Accident, Children, Adolescents, Screening, STEPP

## Abstract

**Background:**

Children and their parents are at risk of posttraumatic stress disorder (PTSD) following injury due to pediatric accidental trauma. Screening could help predict those at greatest risk and provide an opportunity for monitoring so that early intervention may be provided. The purpose of this study was to evaluate the Screening Tool for Early Predictors of Posttraumatic Stress Disorder (STEPP) in a mixed-trauma sample in a non-English speaking country (the Netherlands).

**Methods:**

Children aged 8-18 and one of their parents were recruited in two academic level I trauma centers. The STEPP was assessed in 161 children (mean age 13.9 years) and 156 parents within one week of the accident. Three months later, clinical diagnoses and symptoms of PTSD were assessed in 147 children and 135 parents. We used the Anxiety Disorders Interview Schedule for DSM-IV - Child and Parent version, the Children’s Revised Impact of Event Scale and the Impact of Event Scale-Revised. Receiver Operating Characteristic analyses were performed to estimate the Areas Under the Curve as a measure of performance and to determine the optimal cut-off score in our sample. Sensitivity, specificity, positive and negative predictive values were calculated. The aim was to maximize both sensitivity and negative predictive values.

**Results:**

PTSD was diagnosed in 12% of the children; 10% of their parents scored above the cut-off point for PTSD. At the originally recommended cut-off scores (4 for children, 3 for parents), the sensitivity in our sample was 41% for children and 54% for parents. Negative predictive values were 92% for both groups. Adjusting the cut-off scores to 2 improved sensitivity to 82% for children and 92% for parents, with negative predictive values of 92% and 96%, respectively.

**Conclusions:**

With adjusted cut-off scores, the STEPP performed well: 82% of the children and 92% of the parents with a subsequent positive diagnosis were identified correctly. Special attention in the screening procedure is required because of a high rate of false positives. The STEPP appears to be a valid and useful instrument that can be used in the Netherlands as a first screening method in stepped psychotrauma care following accidents.

## Background

Despite the fact that accidents are widespread, systematic attention for the psychological consequences of accidents is still not common practice. Children who have been injured due to accidental trauma and their parents are at risk of posttraumatic stress disorder (PTSD) [1-3]. PTSD can cause many symptoms that can be grouped into three clusters: 1) re-experiencing symptoms such as flashbacks or nightmares, 2) avoidance symptoms such as avoiding locations, events or other reminders of the experience, 3) hyperarousal symptoms such as sleep or concentration problems or defiant behavior [4,5]. These symptoms disappear spontaneously in the majority of the children, but up to 37.5% develop full or partial PTSD following motor vehicle accidents or unintentional injury [6,7]. PTSD is a debilitating psychiatric disorder, often involving the development of co-morbid disorders [8]. If left untreated, PTSD negatively affects children’s functioning and physical recovery from injury [6].

In the Netherlands, 240,000 children per year are injured in an accident and are subsequently treated in the Emergency Department of a hospital [9]. Medical aftercare following accidents is well organized, but until now no systematic monitoring of the psychological well-being of these children has been available during hospitalization or after discharge.

Post-trauma psychological problems of parents are thought to play a role in the prediction and development of child PTSD [3,10-12]. Parental symptoms can impact child symptoms in various ways. For effective coping assistance, accurate parental judgment is necessary, but the parents’ own symptoms may influence how they judge their child’s needs [13]. Parents with posttraumatic stress symptoms may be less able to support their child [10,14]. Moreover, parents’ symptoms have been found to increase the risk of their child developing PTSD [11]. Following injury to their child, parents are at risk for developing substantial posttraumatic stress symptoms [15]; approximately 15% of the parents develop partial or full PTSD following pediatric injury [16]. Therefore, parents should also be monitored following their child’s accident.

**Figure 1:**
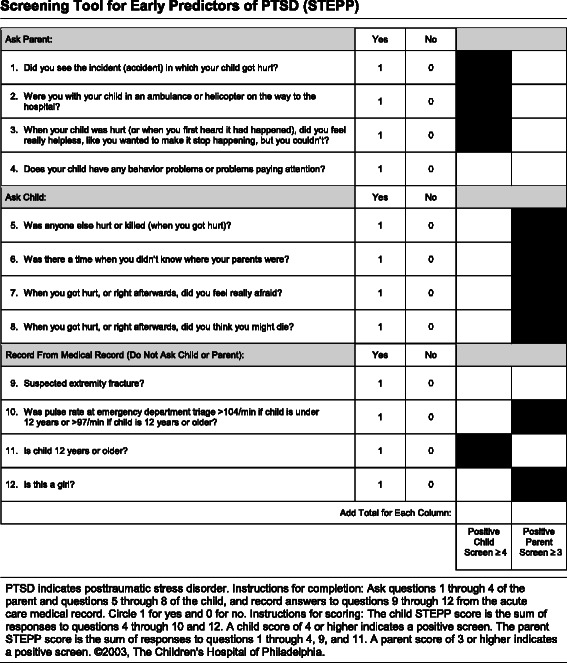
Screening Tool for Early Predictors of PTSD (STEPP).

Identifying children and parents at risk of PTSD creates an opportunity to monitor them. A system of stepped care, offering timely treatment if needed, can contribute to the prevention of chronic trauma-related disorders. For this purpose, Winston and colleagues developed the Screening Tool for Early Predictors of PTSD (STEPP), see Figure 1 [1]. The STEPP appeared to be effective in identifying those who are at risk of persistent posttraumatic stress – both children and their parents – following traffic-related injury to children. Since the purpose of the screening is to identify children and parents who are at risk of PTSD, a high sensitivity is required, while those who are unlikely to develop PTSD should be screened out with a high negative predictive value [1]. STEPP sensitivity in predicting posttraumatic stress was 0.88 for children and 0.96 for parents, with negative predictive values of 0.95 for children and 0.99 for parents [1]. For a further description of STEPP performance, see *Measures*.

However, in an Australian mixed-trauma sample (all single-incident trauma), the STEPP was no better than chance at identifying positive PTSD status in children at either 3 months or 6 months posttrauma. At 3 months, sensitivity of the original STEPP in the Australian sample was 0.45, specificity was 0.68, with a positive predictive value of 0.17 and a negative predictive value of 0.89. An Australian version of the STEPP for children was then compiled from the 8 best performing items in the original item pool of Winston et al. [1]. This Australian STEPP (STEPP-AUS) performed well at 3-months posttrauma: sensitivity was 0.73, specificity was 0.69, with a positive predictive value of 0.26 and a negative predictive value of 0.94. Best performance was at 6 months posttrauma: sensitivity was 0.73, specificity was 0.69, with a positive predictive value of 0.26 and a negative predictive value of 0.94 [17]. Until now, the STEPP has not been validated in other languages or other broader trauma samples.

The purpose of this study was therefore to determine the reliability and predictive performance of the Dutch version of the STEPP in a mixed-trauma sample. If sufficiently predictive, then screening for risk of PTSD would be an effective method to identify those who are at risk: children as well as their parents. In our study we expanded the scope to include unintentional injury in general; we believe it is important to evaluate the STEPP for all types of accidents, not just traffic-related ones.

## Methods

### Participants

Children 8 to 18 years were eligible for inclusion if they survived an accident, were subsequently transported to the hospital by ambulance and underwent a trauma screening in the trauma resuscitation room (trauma room) of the Emergency Department. The standard trauma room procedure was as follows: a multi-disciplinary team of medical specialists and nurses made the initial assessment of trauma patients and provided the initial treatment. Patients were referred to the trauma room in cases with a high-energy trauma mechanism involving a risk of severe and/or potentially life-threatening injuries. Excluded were children who were living abroad at the time of the accident, who stayed on Intensive Care Units (pediatric or regular) for more than one week (the inclusion period) or who were incapable of answering the questions or completing the questionnaires due to cognitive limitations.

We used the trauma registry systems of the Trauma Surgery and Emergency Departments to identify children eligible for this study. We usually invited children to participate in the study via their parents. One parent of each child was also invited to participate. If children had already been discharged, we phoned and asked for an appointment at home. If children were hospitalized, we first consulted the responsible nurse. In total, 266 children were eligible to participate in the study, of which 105 did not participate (26 could not be contacted, 68 declined to participate, and 11 could not be included in time). The final sample consisted of 161 children and 156 parents.

### Procedures

This study was performed at two academic hospitals in Amsterdam, the Netherlands: Academic Medical Center (AMC) and VU Medical Center (VUmc), both Level I trauma centers. The study was approved by the Medical Ethical Committees of both hospitals. Two researchers (EM, MRG), both psychologists, were involved in the study. One of the researchers explained the study to the children and parents, provided them with written information about the study and obtained informed consent. Inclusion was possible only after written informed consent. According to Dutch law, for a child 8-12 years, the parents decide; 12-16 years, parent and child both have to give consent; 16 years and older, the child decides and can give consent autonomously. The inclusion period was between September 2008 and January 2011.

Screening for risk of PTSD was performed within one week of the accident. The STEPP was developed for use in the acute care setting and for assessment by trained nurses. However, this design was not compatible with the routine procedures of the Emergency Departments where we performed the research. For this reason, and to be able to include children who were discharged immediately after the medical screening, assessment was performed by the two researchers. After a general introduction it took about 5 minutes to administer the STEPP questionnaire. To determine intra-rater reliability, the STEPP was assessed twice in a sample of 20 children and 19 parents. When designing the study, we decided to re-administer the STEPP in the second year of the inclusion period to the first 20 children who were discharged. The second assessment was by telephone, within two days after the first assessment. Three months after the accident, PTSD was assessed in an interview conducted at the department of child and adolescent psychiatry in one of the two hospitals. Two different clinically trained psychologists interviewed the child and parent separately. All interviews were audiotaped. Self-report questionnaires were usually completed at home, and in a few cases during the consultation.

To evaluate injury and trauma-related characteristics of the sample, data on duration of hospital stay, trauma type and injury severity were obtained from the trauma registry and the medical records. Information on heart rate upon arrival at the Emergency Department and the type of injury were required to complete the STEPP screening score; this information was obtained from the medical records after finishing the last assessments.

### Measures

#### Injury severity and trauma-related characteristics

The Injury Severity Score (ISS) [18] was obtained from the trauma registry. The ISS is a method for describing the severity of injuries in trauma patients. It is related to the likelihood of survival after injury. The ISS is determined by rating the severity of each injury in six body areas (head, neck, face, chest, abdomen, extremity and external) on the six-point Abbreviated Injury Scale (AIS). The ISS is derived from the sum of the squares of the AIS score and has a range of 0-75 [10,18]. Information on length of hospitalization and trauma type was obtained from the medical records.

#### Screening for risk of PTSD

The STEPP (see Figure 1) is a theoretically derived, empirically validated, stand-alone screening tool [1]. It consists of 12 questions: 4 questions are asked of the child, 4 questions are asked of the parent and 4 items are obtained from the medical records. Including the items from the medical records, the total score for children is based on 8 items, and the total score for parents is based on 6 items. The items are answered dichotomously with “yes” (=1) or “no” (=0). A score of 4 or higher for children and 3 or higher for parents results in a positive screening [1]. For children, the STEPP has shown a sensitivity of 0.88, a specificity of 0.48, a positive predictive value of 0.25 and a negative predictive value of 0.95. For parents, the STEPP has shown a sensitivity of 0.96, a specificity of 0.53, a positive predictive value of 0.27 and a negative predictive value of 0.99. Test-retest reliability was excellent for children (κ = 0.86) and very good for parents (κ = 0.67) [1].

After acquiring permission from the authors, the STEPP was translated into Dutch and then back-translated by a native English speaker. The authors informed us in detail about using, scoring and interpreting the STEPP.

#### Diagnosed children’s posttraumatic stress disorder

To diagnose PTSD in children we used the Dutch version of the Anxiety Disorders Interview Schedule for DSM-IV - Child and Parent Version (ADIS-C/P) [19,20] with an extended adaptation of the PTSD module, including detailed information on trauma history (Verlinden E, van Meijel EPM, Lindauer RJL: Extended version of the PTSD module of the ADIS-C/P, unpublished). The ADIS-C/P is a commonly used diagnostic, semi-structured interview for the assessment of anxiety disorders – including PTSD – and mood and behavioral disorders in children aged 7-17 years. The ADIS-C/P has previously been reported to have good to excellent results regarding test-retest reliability for specific diagnoses (κ = 0.61-1.00) and inter-rater reliability (κ = 0.65-1.0) [21,22]. For a random sample of children in our study (12%), the audiotaped ADIS child and parent interviews were rated independently for inter-rater reliability. The result showed almost perfect agreement (κ = 0.88). The ADIS-C/P showed good reliability for the current sample. Cronbach’s alphas for ADIS-C/P were 0.84 for the child score and 0.80 for the parent score.

Depending on the answer and the clinical interpretation of the interviewer, symptoms can be rated as present or absent. If the number of symptoms endorsed as ‘present’ is enough to meet DSM-IV criteria, impairment in daily functioning is rated on a 9-point Likert scale (0-8). A diagnosis of PTSD requires an impairment level of 4 or more and depends also on the clinician’s judgment of clinical severity. The diagnosis can be based upon either the child report (C) or the parent report (P). The interview also provides for a combined diagnosis, based on both the child and parent report. In cases of disagreement between the two interviews, the child receives a diagnosis if one of the two interviews yields a diagnosis. Partial PTSD is diagnosed when at least one symptom is present in each of three subscales – re-experiencing, avoidance and hyperarousal – resulting in substantial distress or impairment in one or more areas of functioning [1].

The interviewers were extensively trained on administering and scoring the ADIS-C/P and were supervised by an experienced child and adolescent psychiatrist (RJL). The interviewers were blind to the outcome of the STEPP screening.

#### Self-reported children’s posttraumatic stress symptoms

The children completed the Dutch version of the Children’s Revised Impact of Event Scale (CRIES) [23-25]. This self-report measure gives a good indication of the presence of PTSD. It consists of 13 questions in the subscales re-experiencing, avoidance and hyperarousal, with answers on a 4-point Likert scale. Items are rated according to the frequency of their occurrence during the past week (Not at all = 0, Rarely = 1, Sometimes = 3 and Often = 5; range 0-65). We asked the children to focus on their accident when answering the questions. The validation and reliability of the Dutch version of the CRIES was evaluated by Verlinden et al. [25]. Children with PTSD had significantly higher scores than children without PTSD on the total scale of the CRIES (mean score 42.48 versus 19.4; *p* < .001). At a cut-off score of 30, the Dutch CRIES was significantly better than chance at identifying PTSD as measured by the ADIS (area under the ROC curve = 0.91; 95% CI, 0-88-0.94). The CRIES showed excellent test-retest reliability (κ = 0.85) and good reliability: Cronbach’s alpha for the total score was 0.89 [25]. For the current sample Cronbach’s alpha was 0.87. The CRIES showed good agreement with the ADIS-C/P for the current sample. On the CRIES, 16% of the children scored positively; on the ADIS-C/P, 12% of the children were diagnosed with PTSD. These percentages were not significantly different from each other based on the results of the McNemar test of dependent proportions (*p* = .18).

#### Self-reported parental posttraumatic stress symptoms

The parents completed the Dutch version of the Impact of Event Scale-Revised (IES-R) [26,27]. The IES-R consists of 22 questions and contains the subscales re-experiencing, avoidance and hyperarousal. Scoring is on a 5-point Likert scale. Items are rated according to the frequency of their occurrence during the past week (Not at all = 0, A little bit = 1, Moderately = 2, Quite a bit = 3, Extremely = 4; range 0-88). The focus is on the child’s accident. A total score of 23 or above indicates the likely presence of PTSD [28]. The Dutch IES-R showed adequate similarity with the total score of the Clinician-administered PTSD scale (CAPS; *r* = .75, *p* < .001) [28-30] and good reliability for the current sample; Cronbach’s alpha was 0.96.

#### Statistical analyses

We used descriptive statistics to summarize the demographic, trauma-related and clinical characteristics of the sample. Differences between participants and non-participants were analyzed with Mann-Whitney tests for age and injury severity and a Pearson Chi-Square test for sex. Differences between those who completed the second assessment and those who dropped out after the first assessment were analyzed with Mann-Whitney tests for age and injury severity, a Pearson Chi-Square test for sex and a t-test for the STEPP scores.

To evaluate the performance of the STEPP at predicting child and parent PTSD, Receiver Operating Characteristic (ROC) curve analyses and cross-tabulations were conducted. An ROC curve analysis represents the changes in accuracy (sensitivity and specificity) with different positivity thresholds, and thus allows determination of the optimal cut-off point in a sample for a clinically optimal discriminative ability of a test. At the lowest cut-off point, all subjects are classified as test-positive (including the diseased), resulting in 100% sensitivity but 0% specificity. On the other end, at the highest cut-off point, all subjects (including the diseased) are classified as non-diseased, resulting in 0% sensitivity and 100% specificity. The area under the ROC curve (AUC) reflects the overall predictive performance of a test. The maximum value is 1, which means a 100% accurate test, whereas an AUC of 0.50 indicates the test does not perform better than chance. We used the STEPP score as the index test. Diagnosed PTSD and a positive score on the self-report PTSD measures were used as the reference tests. Results of the index test and the reference tests were cross-classified in 2-by-2 tables, and sensitivity, specificity, positive and negative predictive values were calculated. The optimal cut-off score for the STEPP for our sample was based on the decision to maximize both sensitivity and negative predictive values. Intra-rater reliability was tested for the STEPP: the Kappa statistic was used to determine consistency between the first and the second assessment by the same rater.

Statistical analyses were performed using SPSS 18 and 19 (IBM Statistical Product and Service Solutions, Chicago, Ill).

## Results

A total of 161 children and 156 parents completed the first assessment within one week of the accident. Demographic, trauma-related and clinical characteristics of this sample are reported in Table 1. There were no significant differences between participants and non-participants with regard to age (U = 8170, Z = -.467, *p* = .64), sex (χ^2^ = 1.21, *p* = .27) or injury severity (U = 5419, Z = -1.367, *p* = .17).

**Table 1:** **Demographic, trauma-related and clinical characteristics**

	No (%)	Mean (SD)	Min-max
Sex children			
Female	66 (41)		
Male	95 (59)		
Age	161	13.9 (2.8) years	8-17 years
Sex parents			
Female	120 (77)		
Male	36 (23)		
Trauma type			
(Road) traffic accident	115 (71.4)		
Sports accident	20 (12.4)		
Other, including falls	26 (16.2)		
ISS		6.8 (7.7)	0-43
Admitted to hospital	113 (70)		
Days in hospital		4.9 (6.1)	<1-33
Admitted to (P)ICU	22 (14)		
Days on (P)ICU		1.8 (1.5)	<1-6

ISS - Injury Severity Score, (P)ICU - (Pediatric) Intensive Care Unit.

Three months after the accident, 146 children and 139 parents completed the second assessment. Those who dropped out after the first assessment did not differ significantly from those who completed the second assessment in terms of age (U = 908, Z = -.736, *p* = .46), injury severity (U = 939, Z = -.429, *p* = .67), sex (χ^2^ = .02, *p* = .88) or STEPP score (t(159) = -1.92, *p* = .06).

### Posttraumatic stress

PTSD interview-based data were available for 147 children. A combined child/parent informed diagnosis was made for 135 children. For one child, a diagnosis was derived only from the parent report, and for 11 children only from the child report. With the ADIS-C/P, 17 children (11.6%) were diagnosed with PTSD, 9 of them with full PTSD (6.1%) and 8 of them with partial PTSD (5.4%).

A total of 144 children completed the self-report measure CRIES (mean score = 15.67, SD = 13.41). The scores of 23 children (14.3%) were above the cut-off score, indicating serious posttraumatic stress symptoms (mean score = 39.91, SD = 8.16).

In total, 135 parents completed the IES-R (mean score = 9.39, SD = 13.64). Of this group of parents, 13 (9.6%) scored 23 or above (mean score = 45.23, SD = 15.48) which indicates the likely presence of PTSD.

### Performance of the STEPP

The STEPP showed moderate discriminative ability for child PTSD, with areas under the curve for diagnosed PTSD of 0.68 (95% CI 0.53-0.82) and for self-reported PTSD symptoms 0.69 (95% CI 0.56-0.81). The parent score resulted in an AUC of 0.59 (95% CI 0.43-0.75) for self-reported PTSD symptoms, which is too low to discriminate. Results of the ROC analyses are presented in Table 2, showing the accuracy (sensitivity and specificity) and the positive and negative predictive values for different cut-off values for the STEPP. Because a screening instrument should basically identify all cases (maximize sensitivity), the STEPP showed optimal performance in detecting children and parents with PTSD at a cut-off value of 2. High negative predictive values should screen out those who are unlikely to develop PTSD. We therefore had to accept poor specificity, which could lead to false positives.

**Table 2:** **Performance of the STEPP in predicting PTSD at 3 months, at different cut-off scores**

Cut-off	Sensitivity (95% CI)	Specificity (95% CI)	PPV (95% CI)	NPV (95% CI)
Child diagnosis (ADIS-C/P)				
2	0.82 (0.57-0.96)	0.28 (0.20-0.36)	0.13 (0.07-0.21)	0.92 (0.79-0.98)
3	0.65 (0.38-0.86)	0.62 (0.53-0.70)	0.18 (0.09-0.30)	0.93 (0.85-0.97)
4	0.41 (0.19-0.67)	0.87 (0.80-0.92)	0.29 (0.13-0.51)	0.92 (0.86-0.96)
Child self-report (CRIES)				
2	0.87 (0.66-0.97)	0.29 (0.21-0.38)	0.19 (0.12-0.28)	0.92 (0.79-0.98)
3	0.61 (0.39-0.80)	0.62 (0.53-0.71)	0.23 (0.13-0.36)	0.89 (0.81-0.95)
4	0.43 (0.23-0.65)	0.89 (0.82-0.94)	0.43 (0.23-0.65)	0.89 (0.82-0.94)
Parent self-report (IES-R)				
2	0.92 (0.64-0.99)	0.21 (0.14-0.30)	0.11 (0.06-0.19)	0.96 (0.81-0.99)
3	0.54 (0.25-0.81)	0.57 (0.48-0.66)	0.12 (0.05-0.23)	0.92 (0.84-0.97)
4	0.23 (0.05-0.54)	0.88 (0.81-0.93)	0.17 (0.04-0.41)	0.91 (0.85-0.96)

STEPP - Screening Tool for Early Predictors of PTSD, PTSD - Posttraumatic Stress Disorder, AUC - Area Under the Curve, CI - Confidence Interval, PPV - Positive Predictive Value, NPV - Negative Predictive Value, ADIS - Anxiety Disorders Interview Schedule-Child/Parent, CRIES - Children’s Revised Impact of Event Scale, IES-R - Impact of Event Scale-Revised.

Intra-rater reliability was tested for a categorical score (‘At risk’ or ‘not at risk’) based on the cut-off score. At the original cut-off scores (4 for children and 3 for parents), intra-rater reliability showed moderate agreement for both the child and parent part (κ = 0.46 and 0.45 respectively). The differences in answering question 4 (“Does your child have any behavior problems or problems paying attention?”) and question 7 (“When you got hurt, or right afterwards, did you think you might die?) were responsible for two additional cases with positive scores at the second assessment. We found no systematic pattern of discrepancy between test and re-test assessment for either of the items. In one of the cases, even question 2 (“Were you with your child in an ambulance or helicopter on the way to the hospital?”) was answered differently. When using the adjusted cut-off scores of 2, intra-rater reliability improved to substantial for the child part (κ = 0.66) and to almost perfect for the parent part (κ = 0.83).

## Discussion

In a large mixed-trauma sample, we determined that the Dutch version of the STEPP is reliable and predictive. At the originally recommended cut-off scores, the performance of the STEPP in the study of Winston and colleagues was not replicated; the STEPP appeared to perform only moderately in our sample [1]. However, adjusting the cut-off scores improved the predictive performance substantially: 82% of the children and 92% of the parents at risk were correctly identified. This high sensitivity supports the use of the STEPP as a screening tool. The high negative predictive values make the STEPP useful to screen out those who are least likely to develop PTSD. Lower positive predictive values are consistent with the results of other studies and may be a consequence of the low prevalence of PTSD in our sample (11.6% in children, 9.6% in parents) [1,17].

There are several possible explanations for the deviant performance of the STEPP when using the originally recommended cut-off scores. First, in our study we used different measures and a different time frame than in the study of Winston and colleagues [1]. In the latter study, the CAPS-CA was used for the assessment of PTSD, while in our study we used the ADIS-C/P. Winston and colleagues administered the STEPP within one month of the accident, and assessment of PTSD was 3 to 13 months after the accident [1]. In our study we administered the STEPP within one week of the accident and assessment of PTSD was 3 months after the accident. As a consequence, children and parents with delayed onset of PTSD were not included in our study. Furthermore, the STEPP was originally developed in a sample of children who were injured in traffic accidents. In our study we included children who were injured in all types of accidents; it is possible that the various types of accidents have a different impact on the children and parents.

The results of our study are in line with the results of the study of Nixon et al., who compared the effectiveness of various screening instruments following accidental injury in an Australian mixed-trauma sample [17]. As in our study, the STEPP did not accurately predict PTSD in the Australian sample using the original cut-off scores. Because the Australian colleagues at the same time wished to reduce the screening time and effort by not using items from hospital files, they developed a new, alternative screening instrument for children, the STEPP-AUS [17].

Although the results of our study are promising, there is still a challenge for improvement and future research. It would be interesting to investigate the possibilities and benefits of alternative methods to administer the STEPP, for instance by telephone or online. This might be interesting particularly if children are discharged from the hospital immediately after treatment at the Emergency Department.

There are also a few limitations of our study to mention. First, the performance of the STEPP with adjusted cut-off scores requires replication in a larger and independent sample to improve the generalizability. Second, an inherent limitation of the STEPP is its lack of specificity combined with high sensitivity. If used in practice, too many children and parents will therefore need monitoring. This is a potential disadvantage in terms of healthcare costs and may negatively influence the possibilities of implementing the instrument. In a future stepped care model this disadvantage can be addressed by using a brief questionnaire like CRIES or IES-R to determine if children or parents probably have developed PTSD. Only in case of a positive screen would they be referred to further screening and diagnostics. False positive screenings increase the necessity to act very carefully when introducing and supporting the screening procedure. Screening for risk or for symptoms is often seen as an intervention; the challenge is to use the screening procedure in a way that it is supportive for children and parents.

## Conclusions

Screening and monitoring children and parents at risk, preferably integrated in hospital care, can contribute to the prevention of chronic PTSD after accidental injury. A stepped model of psychotrauma care will – in a timely fashion – benefit people who are likely to develop PTSD. Although further improvement and research are needed, a screening tool like the STEPP can be a useful instrument in the first phase of stepped care in the Netherlands.
